# GaAs Solar Cells Grown Directly on V-Groove Si Substrates

**DOI:** 10.1021/acsami.4c18928

**Published:** 2024-12-18

**Authors:** Theresa E. Saenz, Jacob Boyer, John S. Mangum, Anica N. Neumann, Jennifer Selvidge, Sarah A. Collins, Michelle S. Young, Steven W. Johnston, Myles A. Steiner, Ryan M. France, William E. McMahon, Jeramy D. Zimmerman, Emily L. Warren

**Affiliations:** † National Renewable Energy Laboratory, Golden, Colorado 80401, United States; ‡ Department of Physics, Colorado School of Mines, Golden, Colorado 80401, United States

**Keywords:** semiconductors, epitaxy, dislocations, solar cells, III−V, nanopatterning, Si

## Abstract

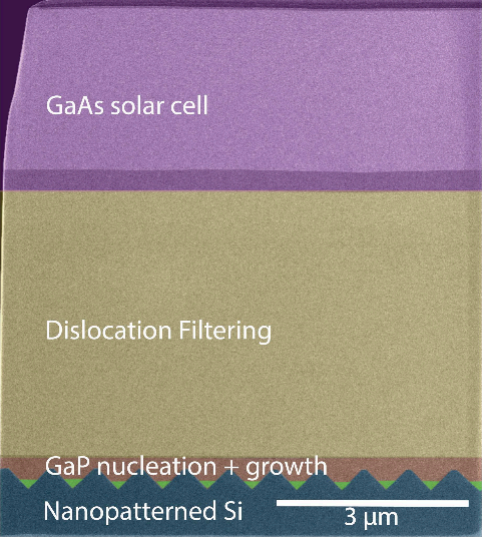

The direct epitaxial growth of high-quality III–V semiconductors on Si is a challenging materials science problem with a number of applications in optoelectronic devices, such as solar cells and on-chip lasers. We report the reduction of dislocation density in GaAs solar cells grown directly on nanopatterned V-groove Si substrates by metal–organic vapor-phase epitaxy. Starting from a template of GaP on V-groove Si, we achieved a low threading dislocation density (TDD) of 3 × 10^6^ cm^–2^ in the GaAs by performing thermal cycle annealing of the GaAs followed by growth of InGaAs dislocation filter layers. This approach eliminates the need for a metamorphic buffer to directly integrate low-TDD GaAs on Si. We used these low-TDD GaAs/V-groove Si templates to grow GaAs double heterostructures that had a minority carrier lifetime of 5.7 ns, as measured by time-resolved photoluminescence, a value consistent with the material quality associated with a 20%+ efficient GaAs solar cell. However, front-junction GaAs solar cells grown on these low-TDD substrates produced a conversion efficiency of only 6.6% without an antireflection coating. Electron channeling contrast imaging measurements on this cell showed a high density of misfit dislocations at the interface between the AlInP/GaInP window layer and the GaAs absorber and between the GaAs absorber and the GaInP back surface field (BSF), likely causing a high surface recombination velocity and thus poor performance. We showed that we could reduce (and in the case of the BSF, eliminate) these dislocations by employing an AlGaAs-based window layer and BSF. Compared to GaInP, AlGaAs has dislocation glide properties that are more similar to those of GaAs, resulting in more even threading dislocation glide between layers. AlGaAs passivation improved the external quantum efficiency and open-circuit voltage of the devices, but the overall device performance was still low at an efficiency of 7.7% without an antireflection coating, likely due to cracking in the devices. This work demonstrates a route to high material quality in GaAs grown directly on Si that can be used for the production of III–V/Si optoelectronic devices.

## 1 Introduction

The integration of high-performance III–V materials with low-cost Si is a potential approach to producing high-efficiency multijunction solar cells,^1^ with mechanically stacked III–V/Si multijunction solar cells demonstrating an efficiency of 35.9%^2^ and bonded III–V/Si multijunction solar cells demonstrating an efficiency of 36.1%.^3,4^ To fully realize the low-cost advantage of Si in a III–V/Si multijunction solar cell, a III–V cell can be grown directly on the Si, thereby eliminating the high cost associated with using a III–V substrate.^5,6^ Achieving efficiencies similar to those of mechanically stacked or bonded III–V/Si multijunction solar cells via direct growth has proven to be difficult. Keeping the minority carrier lifetime of the Si from degrading in the III–V epitaxy reactor environment is a challenge,^7,8^ and differences in material properties between III–Vs and Si can introduce crystalline defects: antiphase domains (APDs) related to the nucleation of lower-symmetry III–Vs onto higher-symmetry Si and high threading dislocation densities (TDDs) related to lattice mismatch, as well as cracking in the III–V films due to a mismatch in the coefficient of thermal expansion between the materials.^9^

Recently, the dominant strategy used to grow III–V solar cells on Si has been to nucleate GaP (nearly lattice matched to Si) on chemo-mechanically polished (CMP), vicinal Si and then grow a GaAs_*x*_P_1–*x*_ step-graded buffer to slowly change the lattice constant to 1.7 eV GaAs_0.75_P_0.25_ (suitable as a tandem solar cell with Si as the bottom junction) or GaAs (suitable for two III–V junctions above the Si, i.e., GaAs/GaInP). Step-graded buffers are well-developed for III–V multijunction solar cells, but they typically benefit from a low TDD at the start of the grade and need to be relatively thick to have a slow grading rate to achieve a low TDD.^10^ Despite the small lattice mismatch, producing low-TDD GaP layers on Si has proven to be challenging. Maintaining a low TDD in the initial GaP growth has been a significant research topic recently,^11,12^ which, with a step-graded buffer used to maintain that low TDD, has enabled higher III–V/Si device efficiencies.^13−15^ Even with this progress, elevated TDD in the III–V films remains a major limiting factor for overall device performance. Earlier work on III–V/Si solar cells using Si_*x*_Ge_1–*x*_-based metamorphic buffer resulted in lower-TDD GaAs than more recent GaAsP-based work, but Si_*x*_Ge_1–*x*_ buffers do not allow for an active Si junction.^16^

In contrast, III–V-on-Si epitaxy research in the on-chip laser community has emphasized dislocation filtering strategies to produce a low TDD in the III–V epilayer.^17,18^ Unlike step-graded buffers, a low dislocation density is not important in the initial III–V layers; materials such as GaAs^19^ with a large lattice mismatch to Si are even used for the first nucleation layer on Si, which is certain to cause high TDD. The exact details of the techniques used vary, but the overarching concept is to induce dislocation glide to both drive dislocation reactions and glide dislocations out of active regions. Thermal cycle annealing (TCA)^20^ and strained superlattices^21^ used as dislocation filtering layers (DFLs) are common strategies. Recently, a newly developed asymmetric step-graded filter combined with thermal cycle annealing achieved a TDD of 2 × 10^6^ cm^–2^ in GaAs in only 2.3 μm of growth on a GaP/Si template.^22^ A TCA/DFL approach has numerous advantages that could be useful for solar cells, but it has seen only limited study in that context.^23−25^

In this work, we implement an asymmetric step-graded filter as a DFL along with TCA to grow GaAs solar cells on GaP/V-groove Si templates by metal–organic vapor phase epitaxy (MOVPE). The approach described here was designed with the cost in mind. The DFL/TCA approach promises to decrease the amount of material needed with the same or lower TDD compared with step-graded buffers. Thinner growth provides a direct cost savings in terms of reactor time and material utilization, and because thick III–V material also drives film cracking,^9^ it also leaves a larger thickness budget for active device regions. Thinner buffers could enable the integration of multiple III–V junctions on Si substrates, which have the potential for even higher device efficiencies. If the III–V multijunction performance is sufficiently high, it could also eliminate the need for an active Si substrate. If the Si does not need to be active, there are far lower requirements for the quality of Si used, allowing for even lower-cost Si: for example, Si from recycled solar panels in a circular economy approach. Additionally, the V-groove Si templates can be fabricated on PV-grade Si substrates, eliminating the cost of the chemo-mechanical polishing typically needed for epitaxy.^26^

## 2 Results and Discussion

### 2.1 Dislocation Filtering

**Figure 1:**
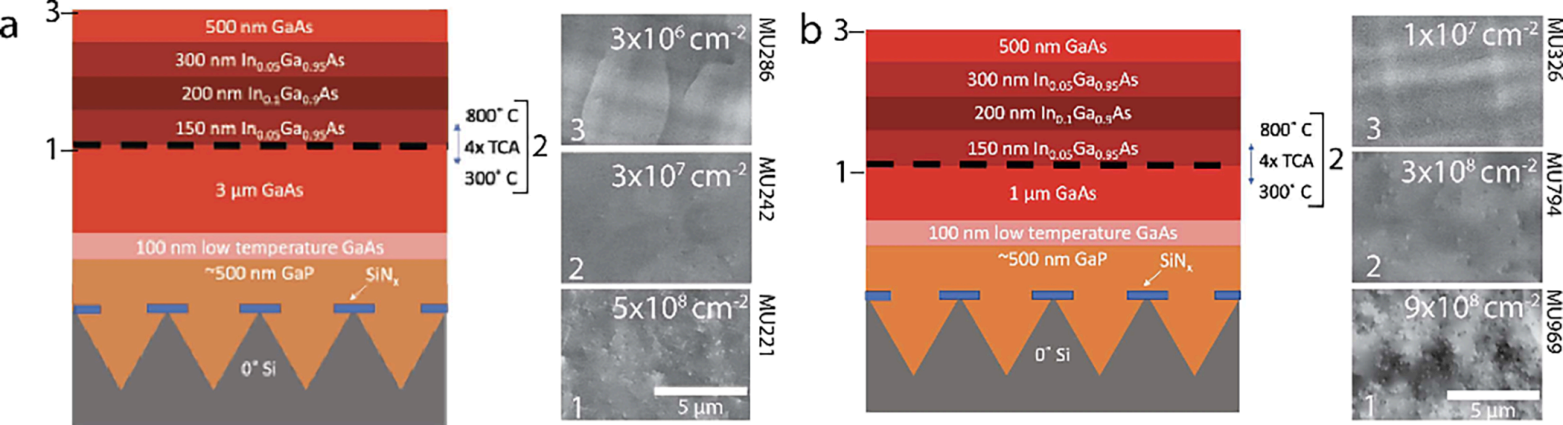
(a) Schematic and ECCI measurements of the structure used to achieve a low dislocation density in GaAs grown on V-groove Si. This structure was used as the template for solar cells and TRPL structures. Plan-view ECCI shows a TDD of (1) 5 × 10^8^ cm^–2^ after 3 μm of GaAs growth, (2) 3 × 10^7^ cm^–2^ after four cycles of TCA between 350 and 800 °C, and (3) 3 × 10^6^ after the growth of an InGaAs dislocation filter structure and a GaAs capping layer. (b) Schematic and ECCI measurements of a control structure with a thinner GaAs buffer resulting in a relatively high TDD. Plan-view ECCI shows a TDD of (1) 9 × 10^8^ cm^–2^ after 1 μm of GaAs growth, (2) 3 × 10^8^ cm^–2^ after four cycles of TCA between 350 and 800 °C, and (3) 1 × 10^7^ cm^–2^ after the growth of an InGaAs dislocation filter structure and a GaAs capping layer.

An initial investigation of a TCA/DFL was carried out on the V-groove Si and GaP templates with several different GaAs buffer thicknesses. For the configuration shown in Figure 1a, the TDD (measured via electron channeling contrast imaging (ECCI)) decreased from 5 × 10^8^ cm^–2^ in the initial GaAs buffer to 3 × 10^7^ cm^–2^ after the TCA, and finally to 3 × 10^6^ cm^–2^ in the GaAs capping layer after the TCA, as shown in the ECCI images in Figure 1. This low TDD is expected to be sufficient for high III–V solar cell performance^27^ and is an improvement over the TDD values achieved in GaAs and GaAsP grown via metamorphic buffers on Si in recent work.^13−15^ We also found that the MOVPE reactor allows for more aggressive TCA conditions than those previously reported by MBE;^22^ the higher AsH_3_ overpressure in MOVPE likely enables higher TCA temperatures than are achievable by MBE. We saw no GaAs surface degradation (i.e., desorption pitting) up to at least 800 °C (and relatively minor degradation at 900 °C) under AsH_3_, as observed by both optical microscopy and scanning electron microscopy (SEM). Other MOVPE-based work reports surface degradation at temperatures of 800 °C,^19^ so the details of the reactor are likely important in determining what temperatures are possible for the TCA. The reactor in this work is a custom-built atmospheric pressure reactor that uses AsH_3_ rather than tertiarybutyl arsine (TBAs). Both of these factors enable a high arsenic overpressure that helps stabilize the GaAs surface at higher temperatures. While we did not explore this variable in this work, MOVPE also should allow greater ranges of In compositions to be used in the DFL layers. The higher growth temperatures accessible by MOVPE reach a window of thermodynamic phase stability for InGaAs, avoiding phase separation issues that are typical for MBE-grown InGaAs for a wider range of In compositions.^28^

The parameter space for optimizing TCA and DFL is large. The number of TCA cycles, the thickness of the GaAs before the TCA, and the thickness, composition, growth temperature, and rate of the DFL are just some examples. While we did not exhaustively explore this parameter space, we did explore several combinations of different GaAs buffer thicknesses and TCA conditions. The resulting TDDs from these experiments are shown in Table 1. It is expected from past theoretical^17^ and experimental^22,29^ results that dislocation density is reduced for a thicker buffer, higher TCA temperature, and larger number of TCA cycles. While we see some hints of these trends, we observe a relatively thick buffer being the key factor for a low TDD. The buffer thickness needed here for a sub-10^7^ cm^–2^ TDD is also greater (3 μm) than that used in Shang et al.^22^ (1 μm). Figure 1b shows a schematic of a DFL structure with a thinner 1 μm GaAs buffer studied in our work, along with ECCI images after the buffer, after the TCA, and after the InGaAs DFL. The initial TDD measured after the 1 μm GaAs buffer is higher than that of the 1 μm GaAs buffer reported in Shang et al.^22^ This higher starting point for TDD reduction may explain why a thicker buffer is required to produce a low-TDD material after the TCA and DFL. It is unclear what causes this higher starting TDD; the elevated TDD in the GaP templates prior to beginning GaAs growth in this work (as opposed to the unrelaxed GaP/Si templates used in Shang et al.^22^), the underlying V-groove Si template, or differences between MOVPE and MBE growth may all have an effect. This topic merits further study, as a well-optimized TCA and DFL would enable a thinner buffer, easing issues with cracking and lowering growth costs. If a thinner, low-TDD buffer can indeed be achieved with MOVPE, as it was in MBE-based work, it would represent a significant reduction in thickness over metamorphic buffers.

**Table 1:** Summary of Dislocation Filter Structures and the Resulting TDD Measured by ECCI under Varying TCA Conditions^a^

GaAs buffer thickness	TCA high temperature	# of TCA cycles	TDD
3 μm	900 °C	4	2 × 10^6^ cm^–2^
1 μm	900 °C	4	1 × 10^7^ cm^–2^
3 μm	800 °C	4	3 × 10^6^ cm^–2^
1 μm	800 °C	4	1 × 10^7^ cm^–2^
1 μm	800 °C	8	2 × 10^7^ cm^–2^
2 μm	800 °C	4	1 × 10^7^ cm^–2^

a The TCA low temperature is always 350 °C. Note that the GaP layer contributes some additional thickness to the overall III–V epitaxy thickness for the TCA. The DFL contributes another 1.15 μm to the final thickness, grown after the TCA.

### 2.2 Time-Resolved Photoluminescence

As an initial assessment of the optoelectronic performance, we grew double heterostructures on the low-TDD GaAs templates as test structures for time-resolved photoluminescence (TRPL) measurements. TRPL extracts a minority carrier lifetime from a symmetric double heterostructure that is a combination of several component lifetimes, as shown in eq 1,^30^ where τ_PL_ is the measured minority carrier lifetime, *B* = 2 × 10^–10^ cm^3^/s and is the radiative recombination coefficient determined by the absorber material, *N*_D_ is the doping level, *D* is the diffusivity of the minority carrier (here, 7.1 cm^2^/s for holes in n-type GaAs^31^), *N*_d_ is the TDD, *S* is the surface recombination velocity (assumed to be the same at both heterointerfaces with the barrier layers), and *d* is the thickness of the test layer.
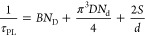
1

Both the second and third terms on the right side of eq 1 are useful for probing the optoelectronic quality of the III–V material grown on Si. The second term, a measure of the bulk nonradiative lifetime, is another way of validating the TDD of the III–V layers. The third term, which is reflective of the quality of the surface passivation of the barrier layers, is useful for understanding the effect of interfacial defects. Misfit dislocations, which are normally associated with lattice mismatch, can form by a second, more recently discovered mechanism in III–V/Si films, even when all of the involved layers are perfectly lattice matched. Kinetically limited misfit dislocations can form upon cooling when threading dislocations glide due to thermal mismatch strain, but they move through certain layers more readily than others.^32^ The effect has been observed between indium-containing layers (resistant to dislocation glide due to solid solution hardening) and gallium-containing layers (less resistant to dislocation glide), as an example.^33^ Should they form, these misfit dislocations are expected to decrease the surface passivation provided by the double heterostructure’s barrier layers. GaInP also has a smaller coefficient of thermal expansion mismatch to Si than GaAs or AlGaAs, meaning that the driving force for dislocation glide (in addition to the kinetic considerations already discussed) is not as large as it is in GaAs.

**Figure 2:**
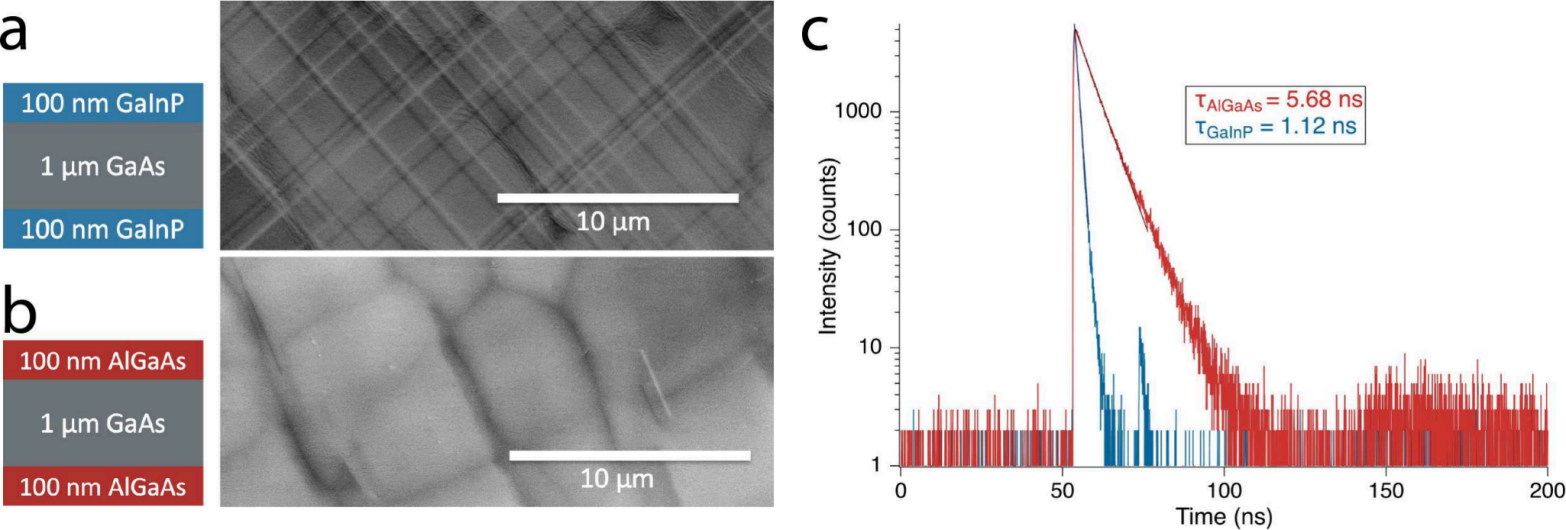
ECCI images of double heterostructures grown on low-TDD GaAs/V-groove Si templates, showing a high density of misfit dislocations at the barrier layer/test layer interface when GaInP barriers are used (a, MU504) but few misfits when AlGaAs is used (b, MU670). The misfit-free AlGaAs sample produced a higher TRPL lifetime. (c) TRPL data of each structure along with the fitted minority carrier lifetimes extracted from the data.

Double heterostructures of GaAs with barrier layers composed of both Ga_0.49_In_0.51_P (expected to have misfit dislocations) and Al_0.5_Ga_0.5_As (not expected to have misfit dislocations, as Al has a similar atomic size to Ga and does not solid-solution harden like GaInP) were grown on the low-TDD GaAs/V-groove Si templates (see Figure 2 for schematics). To study the interfaces for misfit dislocations, we measured the plan-view ECCI on the structures. In a plan-view configuration, ECCI will show misfit dislocations only at the front interface; the back interface is too deep to give any ECCI signal. Baseline double heterostructures were grown on 0° GaAs substrates to calculate a TRPL-predicted TDD. The GaInP and AlGaAs barrier layer baseline double heterostructures had TRPL lifetimes of 36 and 34 ns, respectively. The doping of the absorber layers in both the baseline and test double heterostructures was 2 × 10^17^ cm^–3^, as confirmed with electrochemical capacitance voltage measurements.

ECCI imaging showed that the GaInP-cladded GaAs double heterostructures on the V-groove Si templates had the expected misfit dislocations at the GaInP/GaAs interface (Figure 2a), and the AlGaAs-cladded GaAs double heterostructures had only a few misfit dislocations (Figure 2b). TRPL measures of these structures showed that the AlGaAs-cladded double heterostructure had a minority carrier lifetime of 5.68 ns, compared to 1.12 ns for the GaInP-cladded structure. The difference in minority carrier lifetimes in structures with the same low TDD shows the detrimental effect of the misfit dislocations on the passivation provided by the barrier layers. It is a similar result to that in Fan et al.,^15^ where switching from GaInP barrier layers to AlGaAsP on a III–V/Si step-graded buffer also eliminated interfacial misfit dislocations and resulted in a higher TRPL lifetime. In addition to information about misfit dislocations, it is also possible to extract information about TDD from the TRPL lifetime. Based on eq 1 and the baseline AlGaAs-cladded GaAs TRPL lifetime of 34 ns, the TRPL measurement predicts a TDD for the AlGaAs-cladded GaAs/V-groove Si sample of 2.7 × 10^6^ cm^–2^, in good agreement with the 3 × 10^6^ cm^–2^ measured by ECCI.

A minority carrier lifetime of 5.7 ns compares favorably with literature values for III–V-on-Si double heterostructures. In ref (15), p-GaAs_0.75_P_0.25_ double heterostructures with a similar doping level to the structures grown here on the low-TDD buffer were used to produce 25% GaAsP/Si tandems. The heterostructures had a TRPL lifetime of 1.5 ns, although it should be noted that the TRPL lifetime of p-type material is more adversely affected by threading dislocations than the n-type.^31^ In a different work, using SiGe step-graded buffers, an n-GaAs double heterostructure had a minority carrier lifetime of 7.7 ns,^34^ and GaAs single-junction solar cells grown on a similar template later gave an efficiency of 18.1% and a *V*_OC_ of 0.97 V.^35^ These previous results suggest that the material quality demonstrated here, as confirmed by TRPL and ECCI, is sufficient for a highly efficient GaAs solar cell.

### 2.3 GaAs Solar Cells

**Figure 3:**
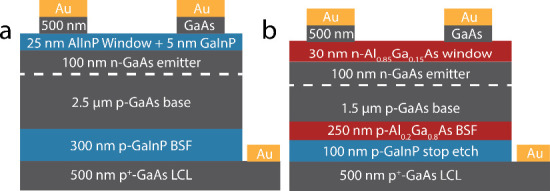
Schematics of (a) the AlInP/GaInP-passivated GaAs solar cells (base doping of 9 × 10^17^ cm^–3^ per capacitance–voltage measurement) and (b) the AlGaAs-passivated GaAs solar cells (base doping of 5 × 10^17^ cm^–3^).

Upright-grown, front-junction GaAs solar cells, both GaInP-passivated and AlGaAs-passivated, were grown by MOVPE on low-TDD GaAs/V-groove Si templates. The GaInP-passivated cell was expected to have misfit dislocations at the window/emitter interface and the back surface field (BSF)/base interface, and the AlGaAs-passivated cell was not. The devices did not have an antireflection coating and were small (500 μm × 500 μm for the AlGaAs-passivated cell and 1.4 mm × 1.4 mm for the GaInP/AlInP cell) due to cracking, which will be discussed in greater detail in Section 2.4. Baseline cells of the same size as those grown on the low-TDD GaAs on V-groove Si templates (small baselines) were grown as a direct comparison to the test cells. Larger baseline cells of 5 mm × 5 mm to test cell quality were also grown of the same structure (Figure 3).

**Figure 4:**
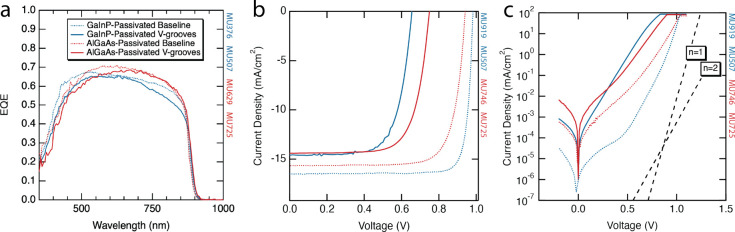
(a) External quantum efficiency of GaInP-passivated and AlGaAs-passivated GaAs/V-groove Si solar cells scaled and compared to corresponding large baseline solar cells grown on GaAs. (b) Light *I*–*V* and (c) dark *I*–*V* curves of the GaInP-passivated and AlGaAs-passivated GaAs/V-groove Si solar cells compared to their corresponding small baselines.

Solar cell results, including the EQE and dark *I*–*V* and light *I*–*V* curves, are shown in Figure 4. Light *I*–*V* parameters (open circuit voltage, short circuit current, fill factor (FF), and efficiency (η)), including those of the large- and small-baseline AlGaAs-passivated and GaInP-passivated solar cells, are summarized in Table 2. The small size of the smaller baseline cells and test samples made the EQE measurement (Figure 4a) artificially low as the EQE spot size was larger than the solar cells. More can therefore be learned from the EQE shape when it is scaled to that of the baseline. EQE shape is not affected by size—perimeter recombination, which can increase recombination current^36^ but is not thought to affect quantum efficiency. We validated this assumption by confirming that the small baseline EQE has the same shape as the large baseline (not shown).

**Table 2:** Summary of Solar Cell Light *J*–*V* Metrics

substrate	passivation	cell size (mm^2^)	*V*_OC_ (V)	*J*_SC_ (mA/cm^2^)	FF	η
GaAs	GaInP	25 (0.3% shading)	1.00	19.8	86%	17.0%
GaAs	GaInP	1.96 (13% shading)	0.98	16.5	85%	13.8%
V-groove Si	GaInP	1.96	0.65	14.6	69%	6.6%
GaAs	AlGaAs	25 (0.3% shading)	1.01	20.3	83%	17.1%
GaAs	AlGaAs	0.2072 (4% shading)	0.94	15.7	77%	13.1%
V-groove Si	AlGaAs	0.2072	0.75	14.4	71%	7.7%

The EQEs of V-groove cells were scaled so that the maximum of the V-groove EQE was the same as the EQE of the baseline at the same wavelength. For the GaInP-passivated cells, the V-groove test sample had some degradation in both long and short wavelengths, suggesting problems with both the window layer and BSF passivation. This result is consistent with the presence of misfit dislocations at those interfaces. For the AlGaAs-passivated cells, some degradation was apparent in the short wavelengths, suggesting problems, particularly with the front of the solar cell. However, the performance at the long wavelengths was much improved, pointing to better passivation from the BSF. Another promising sign in the EQE of both V-groove cells was the lack of a decrease in the EQE at long wavelengths; this is indicative of a sufficiently high minority carrier diffusion length, consistent with a low TDD.

Light *I*–*V* curves of the V-groove solar cell and small baseline cells are shown in Figure 4b. The smaller size alone causes a decrease in open-circuit voltage due to edge recombination^36^ for the baseline cells relative to their large-area counterparts, from 1.01 to 0.94 V for the AlGaAs-passivated cell and from 0.99 to 0.98 V for the GaInP-passivated cell. However, both designs of V-groove cells had much lower open-circuit voltages than the baselines, at 0.65 V for the GaInP-passivated cell and 0.75 V for the AlGaAs-passivated cell. Some, if not all, of this voltage loss is likely caused by cracking in the cells. Dark *I*–*V* curves (Figure 4c) show that both V-groove solar cells had much higher *n* = 2 recombination currents than the baseline. Both crystalline defects and edge recombination contribute to this recombination current. Additionally, there is some short-circuit current loss from the baseline for both V-groove cells. Some of the loss in short-circuit current can be attributed to cracking causing a smaller than expected active area, as observed by the electroluminescence measurements discussed in the next section. It is also important to note that the different sizes of the GaInP-passivated and AlGaAs-passivated cells resulted in significantly different grid shading (13% vs 4%), so comparing the short-circuit current density of the GaInP-passivated vs the AlGaAs-passivated cells is not meaningful.

**Figure 5:**
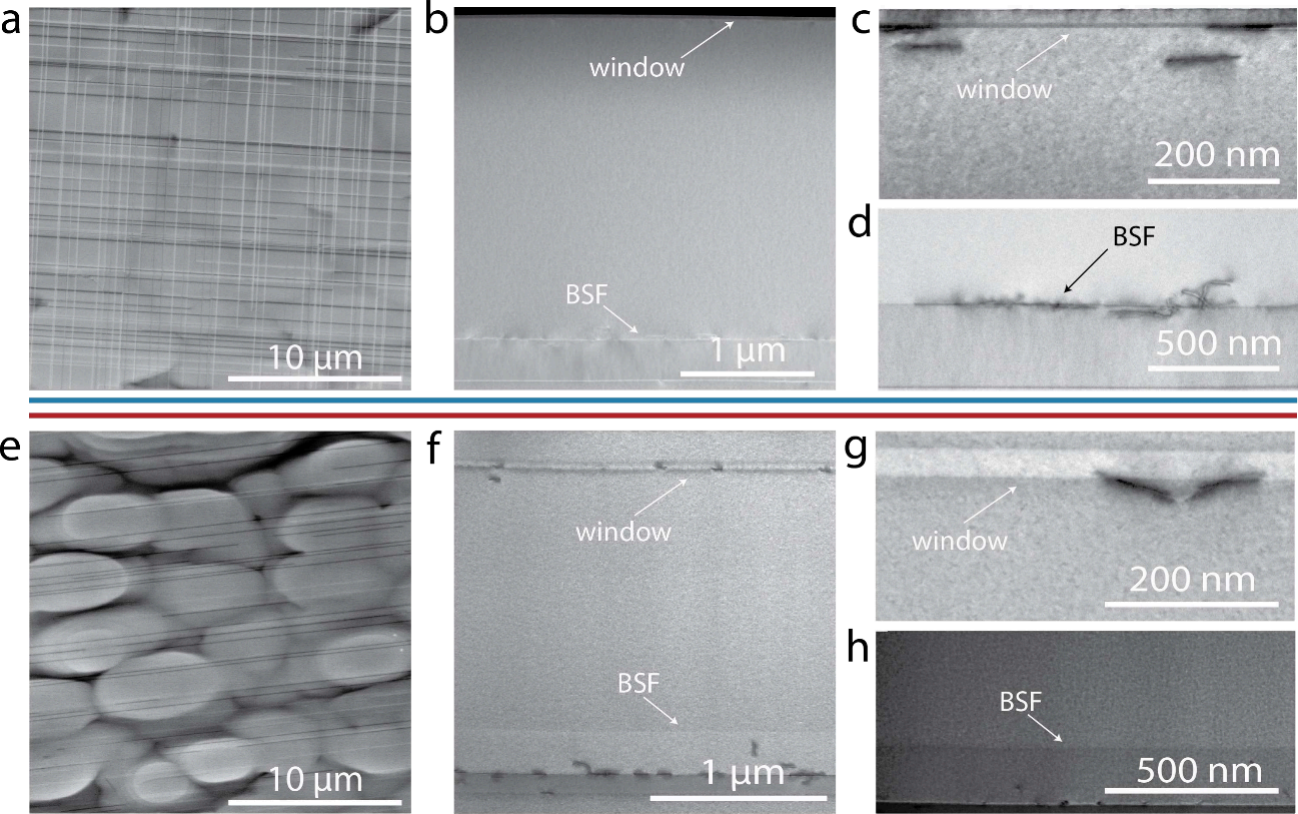
(a) Plan-view ECCI image of a GaInP-passivated GaAs solar cell (all images above blue line; design shown in Figure 3a) grown on V-groove Si showing a high density of misfit dislocations at the window/emitter interface. (b) STEM showing misfit dislocations at the window/base and base/BSF interface in the GaInP-passivated GaAs solar cell. (c and d) Higher-magnification images of these two interfaces. (e) In ECCI of the AlGaAs-passivated cell (all images below red line; design shown in Figure 3b), misfits are still visible, but only in one direction. (f) STEM showing misfit dislocations at the window/base but not the base/BSF interface in the AlGaAs-passivated GaAs solar cell. (g and h) Higher-magnification images of these two interfaces.

ECCI and STEM images of these solar cells show material defects that likely account for at least some the cause of the solar cells’ poor performance. A plan-view ECCI image of the GaInP-passivated solar cell (Figure 5a) shows a high density of misfit dislocations at the window/emitter interface. A cross-section STEM image (Figures 5b and 5c) of the same solar cell confirms the presence of these misfit dislocations but also shows additional dislocations at the base/BSF interface (Figures 5b and 5d). Both of these sets of misfit dislocations were expected; because both the window and BSF are made from In-containing alloys, they are expected to cause the formation of misfit dislocations upon cooling, as was the case for double heterostructures. However, ECCI and STEM imaging also show misfit dislocations in unexpected places. The ECCI image of an AlGaAs-passivated solar cell (Figure 5e) shows misfit dislocations near the top of the solar cell but only running in one ⟨1 1 0⟩ direction instead of both. STEM imaging confirmed that these misfits were at the window/base interface (Figures 5f and 5g) and also showed that there are no misfits at the base/BSF interface (Figures 5f and 5h). This window layer contained a higher percentage of aluminum (85%) than both the BSF (20%) and AlGaAs-based double heterostructures (50%) described earlier. Another solar cell structure was grown with a 50% Al AlGaAs window layer to be more similar to the misfit-dislocation-free double heterostructure, but it too had misfit dislocations at the window layer interface (not shown). It is possible that changes in doping at the p–n junction in the solar cell, something not present in double heterostructures, influences the formation of these misfit dislocations. Dislocation glide velocity is known to depend on doping to differing degrees for α and β dislocations,^37^ so there is a plausible mechanism for a p–n junction to cause the formation of kinetically limited misfit dislocations.

### 2.4 Cracking of III–V Layers on V-Groove Si

**Figure 6:**
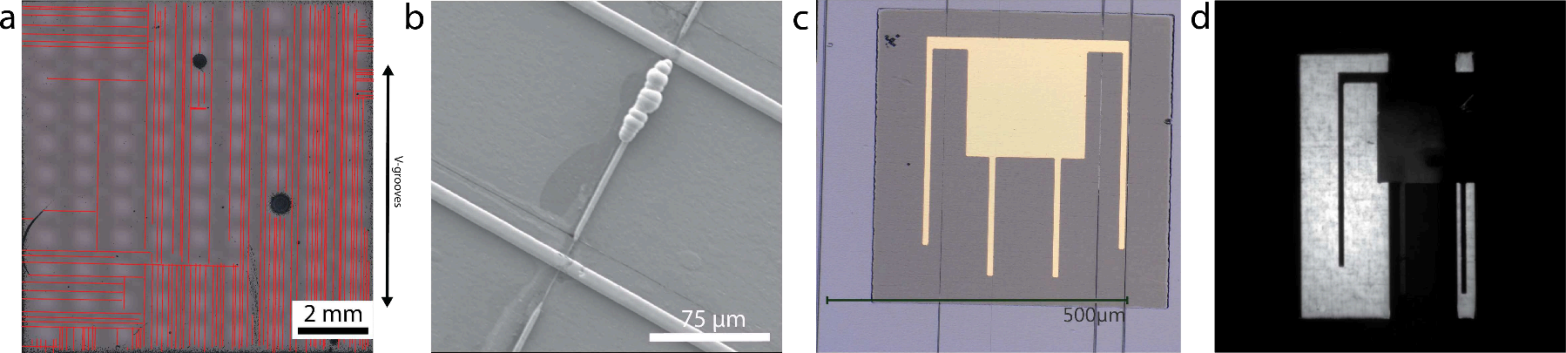
(a) Optical image with cracks highlighted in red showing the crack array on a GaAs on a V-groove solar cell with a 7.4 μm total III–V stack thickness (MU507). (b) SEM image of a crack formed prior to processing that was filled with gold during electroplating, crossing two fingers. Two cracks also run parallel to the fingers, but they formed during processing and are not filled with gold. (c) Optical image of a processed solar cell crossed by several cracks that formed during processing. (d) Electroluminescence image of a GaAs on V-groove solar cell showing significant regions on the cell not working because of cracking.

As has already been alluded to, the devices in this work suffered from cracking due to the coefficient of thermal expansion mismatch between III–V and Si. Table 3 summarizes the crack densities of various structures grown for this work as well as the total growth thicknesses of those structures. An example of an optical image with the cracks highlighted in red is also shown in Figure 6a. With some notable exceptions, the cracks tended to have a higher density in the direction of the V-grooves. Past work has also shown this trend,^23^ and there is also theoretical work showing that V-grooves may act as stress concentrators, potentially exacerbating cracking.^38^ The cracks reported in this table formed when the sample was cooled in the reactor and have to be avoided during cell processing. If these cracks are in the defined cell area before processing begins, they can fill with gold during front contact metallization and shunt the cell, as is shown in Figure 6b. Metal in the cracks completely shunts the solar cell and makes the device immeasurable. For solar cells, we chose areas free of cracks based on optical images to define devices. Despite choosing crack-free areas for cell fabrication, all of the devices still had cracks through them by the end of device processing, such as those seen in Figure 6c. These cracks first became visible after the mesa etch following the front grid metallization. It is unclear if the cracks were already present and widened to be visible during the mesa etch, or if they first formed during processing. They do not correlate with a low shunt resistance in the solar cell, so they do not fill with gold like the cracks that are visible post growth. These cracks either do not form or do not reach the surface until later in processing. The cracks lead to significant portions of the active area not contributing to the photocurrent, as can be seen in the electroluminescence image (taken at 5 mA current) in Figure 6. These dark areas are also a source of increased dark current and thus reduce the open-circuit voltage. Additionally, cracks are expected to increase perimeter recombination,^39^ which leads to a higher nonideal recombination current (*J*_0,2_) causing a lower open-circuit voltage and fill factor.

**Table 3:** Summary of Crack Densities of Devices Grown on a DFL on V-Grooves

run	device structure	total III–V thickness	crack density ∥ V-grooves	crack density ⊥ V-grooves
MU507	GaAs cell, GaInP passivation	7.4 μm	38 cm^–1^	8 cm^–1^
MU703	GaAs cell, AlGaAs passivation	6.4 μm	38 cm^–1^	2 cm^–1^
MU725^a^	GaAs cell, AlGaAs passivation	6.4 μm	2 cm^–1^	48 cm^–1^
MU754	GaAs cell, AlGaAs passivation	5.4 μm	14 cm^–1^	25 cm^–1^
MU504	GaAs DH, GaInP barriers	5.3 μm	76 cm^–1^	1 cm^–1^
MU661	GaAs DH, AlGaAs barriers	5.3 μm	0.4 cm^–1^	7 cm^–1^
MU670	GaAs DH, AlGaAs barriers	5.3 μm	2 cm^–1^	7 cm^–1^
MU286	dislocation filter structure only	4.1 μm	0 cm^–1^	0 cm^–1^

a Slow-cooled.

We attempted to reduce the cracking, guided by recent work showing that dislocation glide during cooldown helps reduce thermal mismatch strain and thus helps prevent cracking.^40^ To allow more time for dislocation glide during cooldown, the sample was cooled at a much lower rate than usual for these samples. The slow cool is intended to allow dislocations to glide further at low temperatures, as their glide velocities decrease at low temperature.^40^ As can be seen in Table 3, this technique did not cause a sizable change in crack density. However, the dominant direction of the cracks changed from parallel to perpendicular relative to the V-grooves, pointing to the possibility that this technique did affect dislocation glide but not symmetrically across each set of threading dislocations (i.e., α vs β dislocations). The influence of dislocation glide on cracking is an important area for further work; one interesting possibility is to enhance dislocation glide during cooling by exposing the sample to light during the cooldown. Such an approach could make use of the recombination-enhanced glide phenomena^41,42^ known for being detrimental to III–V-on-Si laser reliability. Addressing cracking, which has always been a problem, will need to become more of a focus as lower TDD levels are achieved; because dislocation glide helps decrease thermal stress, fewer dislocations available to glide means more residual thermal stress driving the films toward cracking. Indeed, as dislocation density reaches optoelectronics-compatible levels, as demonstrated in this work and others, cracking looks likely to become the dominant challenge facing III–V/Si direct integration efforts.

The clearest trend in the data in Table 3 is a lower crack density for the thinner samples. Less cracking in thinner samples is expected, as a larger thickness produces more thermal mismatch stress.^9^ Notably, the AlGaAs-passivated double heterostructures have a lower crack density than other samples of similar thickness. The lack of misfit dislocations pinned at the interfaces in those samples may allow threading dislocations to glide more during cooldown and relieve more thermal mismatch strain. The thinnest structure (the dislocation filter with no additional device on top) did not crack at all. This result suggests a critical thickness for cracking somewhere between 4 and 5 μm, highlighting the need for better optimization of the TCA and DFL. If the TCA buffer and DFL can be made thinner with the same TDD reduction efficacy, as was the case for MBE-based work,^22^ it will be easier to avoid cracking.

## 3 Conclusion

A TDD of 3 × 10^6^ cm^–2^ was achieved in GaAs grown on GaP/V-groove Si templates through the implementation of an asymmetric step-grade filter and thermal cycle annealing. A minority carrier lifetime of 5.7 ns was demonstrated with a GaAs double heterostructure on this template when AlGaAs barrier layers were used. When using GaInP barrier layers, misfit dislocations formed at the GaInP/GaAs interface, and the minority carrier lifetime was degraded to 1.1 ns. GaAs solar cells grown on these templates had a maximum efficiency of 7.7% (without an ARC). Both cracking and interfacial misfit dislocations are under investigation as potential causes of this low performance. The use of AlGaAs passivating layers did not completely impede the formation of interfacial misfit dislocations in the solar cells; the presence of the misfits likely contributes to their poor performance and is an ongoing subject of investigation. Finally, cracking also likely had a detrimental effect on solar cell performance. Reducing cracking is important to focus on in future work.

## 4 Experimental Section

### 4.1 Sample Preparation and Nucleation

V-groove nanopatterns were fabricated on CMP Si substrates as described in ref (43) by nanopatterning lines onto an SiN_*x*_ hard mask on an exact-oriented (0 0 1) Si wafer, opening the hard mask with reactive ion etching, and selectively etching the exposed Si in KOH to create (1 1 1)-oriented facets. Directly prior to growth, the samples were cleaned with a wet etch of 30 s in 2% HF, 1 min in 4:1 sulfuric acid:hydrogen peroxide piranha etch, and 15 s in 2% HF.

III–V nucleation growths were carried out in a custom-built atmospheric pressure reactor using TMGa, AsH_3_, and PH_3_ as the group V precursors. GaP was nucleated and coalesced over the V-groove nanopatterns with a V/III ratio of 5000 and a *T*_g_ = 800 °C, as described in refs (43) and (44).

### 4.2 Dislocation Filtering

These virtual substrates, which had a TDD of ∼5 × 10^7^ cm^–2^, were then loaded into a second MOVPE reactor equipped with low-temperature-compatible triethylgallium for GaAs growth. The dislocation filter strategy used here was adapted from Shang et al.,^22^ a molecular beam epitaxy (MBE)-based work. First, 100 nm of GaAs was grown at a low temperature (500 °C) to promote smooth, albeit high-TDD, growth, despite the large lattice mismatch between GaAs and GaP. Then, a GaAs buffer layer was grown at 650 °C. This highly defective GaAs was subjected to thermal cycle annealing (TCA) as a first step to reduce the high TDD of the GaAs film. After the TCA, growth resumed, with three InGaAs DFLs grown at 650 °C with the following nominal thicknesses and compositions: 150 nm of Ga_0.95_In_0.05_As, 200 nm of Ga_0.90_In_0.10_As, and 300 nm of Ga_0.95_In_0.05_As, followed by a 500 nm GaAs capping layer. A schematic of this structure is shown in Figure 1. Electron channeling contrast imaging (ECCI)^45^ was used to measure the TDD of the initial GaAs buffer, the same buffer after it was subjected to the TCA, and the final GaAs capping layer above the InGaAs DFL. To ensure statistical significance, at least 100 dislocations were counted on any given sample to determine TDD via ECCI.

### 4.3 Time-Resolved Photoluminsence

All TRPL measurements were made using a 670 nm laser pulsed at 500 kHz and a measured power of 8.7 μW with a spot size of 50 μm. The injection level for these conditions is 9.4 × 10^16^ cm^–3^.^46^ The data were then fit to a single exponential decay model to obtain the TRPL lifetime. Multiple TRPL measurements on any given sample yielded minority carrier lifetimes within 1 ns of each other.

### 4.4 GaAs Solar Cells

Two solar cell structures were tested based on the results of the double heterostructure study. Two designs of solar cell passivation were used: one with passivation layers free of In (Al_0.2_Ga_0.8_As:Zn BSF and an Al_0.85_Ga_0.15_As:Se window layer) and one with passivation layers containing In (lattice-matched GaInP:Zn BSF and lattice-matched GaInP/AlInP:Se window layer). The solar cells had a GaAs:Si+Se front contact layer and a GaAs:Zn lateral conduction layer (LCL) for the back contact. Schematics of these structures are shown in Figure 3. In the case of the AlGaAs-passivated solar cell, a GaInP layer was grown between the BSF and the LCL to act as a stop-etch for the mesa isolation etch. The same solar cell structures were also grown on a 0° (0 0 1) GaAs wafer as references. An Au back contact was electroplated on the lateral conduction layer, and Ni/Au fingers were electroplated as the front grid. The GaAs contact layer was etched away between the grid fingers, and the solar cell was meso-isolated with selective etchants.

External quantum efficiency (EQE) was measured on a custom-built tool using chopped, monochromatic light from a tungsten halogen lamp, as well as a current–voltage preamp and lock-in amplifier, to measure the generated photocurrent. The EQE was then used, in conjunction with a GaAs reference cell, to calibrate the intensity of a Xe lamp on a solar simulator for light *J*–*V* measurements. Error for the overall efficiency measurement of solar cells is expected to be <5% using this procedure. Finally, the solar cell was imaged with plan-view ECCI to observe the crystallographic defects in the final device structure. The solar cells were also studied with ECCI at mixed diffraction conditions and/or with scanning transmission electron microscopy (STEM) for more details on the defects.

### 4.5 Cracking of III–V Layers on V-Groove Si

Crack density after growth was measured using optical imaging on a Keyence VHX-6000 microscope and counting the number of cracks crossing a 1 cm line measured across the sample in the ⟨1 1 0⟩ and ⟨1 1 0⟩ directions. In an attempt to reduce the observed cracking, the cooling rate was reduced. The standard cooling rate is around 70 °C/min from 650 to 250 °C. For the slow cool, the sample was cooled from 650 to 475 °C at a rate of 4 °C/min, held at 475 °C for 20 min, and then cooled from 475 to 350 °C at a rate of 1 °C/min.
